# Transcriptomic Changes During the Replicative Senescence of Human Articular Chondrocytes

**DOI:** 10.3390/ijms252212130

**Published:** 2024-11-12

**Authors:** Aysegul Atasoy-Zeybek, Gresin P. Hawse, Christopher V. Nagelli, Consuelo M. Lopez De Padilla, Matthew P. Abdel, Christopher H. Evans

**Affiliations:** 1 Musculoskeletal Gene Therapy Research Laboratory, Department of Physical Medicine and Rehabilitation, Mayo Clinic, Rochester, MN 55905, USA; 2 Department of Orthopedic Surgery, Mayo Clinic, Rochester, MN 55905, USA

**Keywords:** osteoarthritis, aging, chondrocyte replicative senescence, Hayflick limit, transcriptomics

## Abstract

Aging is a major risk factor for osteoarthritis (OA), but the specific mechanisms connecting aging and OA remain unclear. Although chondrocytes rarely divide in adult articular cartilage, they undergo replicative senescence in vitro, offering a model to study aging-related changes under controlled conditions. OA cartilage was obtained from an 80-year-old male and a 72-year-old female, while normal cartilage was sourced from a 26-year-old male. Chondrocyte cultures were established and sub-cultured to their Hayflick limit. Bulk RNA sequencing on early- and late-passage human articular chondrocytes identified transcriptomic changes associated with cellular aging. Early-passage OA chondrocytes already showed senescent phenotypes, unlike normal chondrocytes. All three cultures underwent 30 population doublings before replicative exhaustion, at which point all cells displayed senescence. During this process, cells lost their ability to form cartilaginous pellets. Differential gene expression analysis revealed distinct transcriptomic profiles between early- and late-passage chondrocytes and between normal and OA-derived cells. Genes related to matrix synthesis, degradation, inflammation, and the senescence-associated secretory phenotype (SASP) showed significant expression changes. Despite being a small pilot study, these findings suggest that further research into the molecular and metabolic changes during chondrocyte senescence could provide valuable insights into OA pathobiology.

## 1. Introduction

Osteoarthritis (OA) is the most common form of arthritis in adults and is characterized by cartilage degradation, joint inflammation, and bone remodeling, which lead to joint pain, swelling, stiffness, and ultimately loss of joint function [1]. The number of people with OA continues to rise, with an estimated 595 million adults affected globally [2]. The knee is the most frequently impacted joint, with a prevalence of 365 million cases, followed by the hip and hand [3]. Different factors such as sex, obesity, genetics, hormone changes, anatomy, and previous joint injury contribute to the development of OA; however, age remains the greatest risk factor for the development of OA [4]. Among people with OA, there are twice as many women as men, especially those with arthritis in the knees. Particularly, the prevalence and risk rapidly increase after menopause (≥45 years) when compared to men of similar age [5,6,7]. Due to the limited treatment options, knee replacement surgery is often the final intervention for patients with OA [8]. As a result, OA represents a significant public health issue and societal burden globally.

Although various different articular tissues undergo collaborative pathological changes in OA, much attention has focused on articular cartilage, whose degeneration is a hallmark of OA. The articular cartilage serves as a low-friction, load-bearing surface, and its breakdown leads to pain, stiffness, and reduced joint function [9]. The degradation of cartilage results from a complex interplay of mechanical stress, inflammation, and molecular changes within the joint [10]. As the cartilage deteriorates, subchondral bone thickening, synovial inflammation, and osteophyte formation often follow, further contributing to the progression of OA [11,12]. Multiple studies of articular cartilage have documented changes resulting from age and OA, including enhanced degradation of components of the extracellular matrix (ECM), particularly aggrecan (*ACAN*), the major proteoglycan of cartilage, and declines in chondrocyte cell yields [13,14]. Such changes contribute to cartilage thinning, mechanical weakening, and, ultimately, loss of cartilage function [15]. Furthermore, the mechanical and frictional properties of articular cartilage undergo significant alterations during OA progression, affecting the tissue’s ability to withstand continuous loading during joint movement. OA induces substantial changes in both the macro- and microstructural organization of cartilage, which in turn disrupts its mechanical integrity. Krakowski et al. addressed these alterations in the mechanical and frictional properties of cartilage during OA progression and provided a comprehensive overview of the mechanical outcomes of various cartilage repair techniques [16].

Chondrocytes derived from older individuals exhibit characteristic features of cellular senescence, including shortened telomeres, heightened cell mortality, elevated senescence-associated β-galactosidase (SA-β-Gal) activity and increased expression of key regulators such as *p53*, *p21^CIP1^*, and *p16^INK4A^* [17,18]. Moreover, they produce increased amounts of reactive oxygen species (ROS) known to trigger the activation of genes associated with chondrocyte dedifferentiation and senescence [19]. Senescent chondrocytes adopt a senescence-associated secretory phenotype (SASP) [20,21]. This involves the secretion of pro-inflammatory cytokines [22], including interleukins (*IL-*), oncostatin M (*OSM*), granulocyte–macrophage colony-stimulating factor (*GM-CSF*), and tumor necrosis factor-alpha (*TNFα*), as well as matrix metalloproteinases (*MMPs*) [23,24] and other proteinases that degrade the ECM of cartilage, including members of the “a disintegrin and a metalloproteinase with thrombospondin motifs” (*ADAMTS*) family. There is also altered activity and expression of growth factors [25,26,27,28], including transforming growth factor-beta (*TGF-β*) and insulin-like growth factor-1 (*IGF-1*). Notably, there is evidence of elevation in SASP factor levels with age in OA patients [29,30]. These age-related alterations in cartilage physiology are likely contributors to the onset and progression of OA.

As well as undergoing aging in situ, chondrocytes undergo replicative senescence when serially passaged in vitro. The irreversible cell cycle arrest that occurs after a finite number of cell divisions in vitro was first described by Hayflick and Moorhead [31,32]. Primary human fibroblasts in culture underwent a progressive loss of mitotic capacity with repeated passaging, leading to complete mitotic arrest after approximately 30–40 population doublings. The inability of primary cells to proliferate beyond a finite number of doublings is now known as the “Hayflick limit”. Evans and Georgescu [33] first demonstrated that cultures of articular chondrocytes from rabbits, dogs, and humans undergo replicative senescence and suggested that chondrocyte senescence explains the association of OA with age. Because chondrocytes in adult cartilage rarely divide, even in OA, it was suggested that aging in situ reflected extended periods in the G_0_ phase of the cell cycle [34]. Senescent chondrocytes in articular cartilage were shown to accumulate with age from human OA samples [35]. Moreover, the transplantation of senescent cells into the knee joints of wild-type mice induced an OA-like condition characterized by pain, impaired mobility, and both morphological and histological changes [36]. Multiple review articles have discussed the role of chondrocyte senescence in the initiation and progression of OA [37,38,39].

In this pilot study, we studied chondrocytes from the knees of three individuals: two older individuals (72 and 80 years old) with OA and one young adult (26 years old) without OA. Chondrocytes from all donors were serially passaged to replicative senescence [33], and bulk RNA sequencing was performed to identify passage-related transcriptomic changes.

## 2. Results

### 2.1. Histology and Replicative Senescence of Chondrocytes

**Figure 1:**
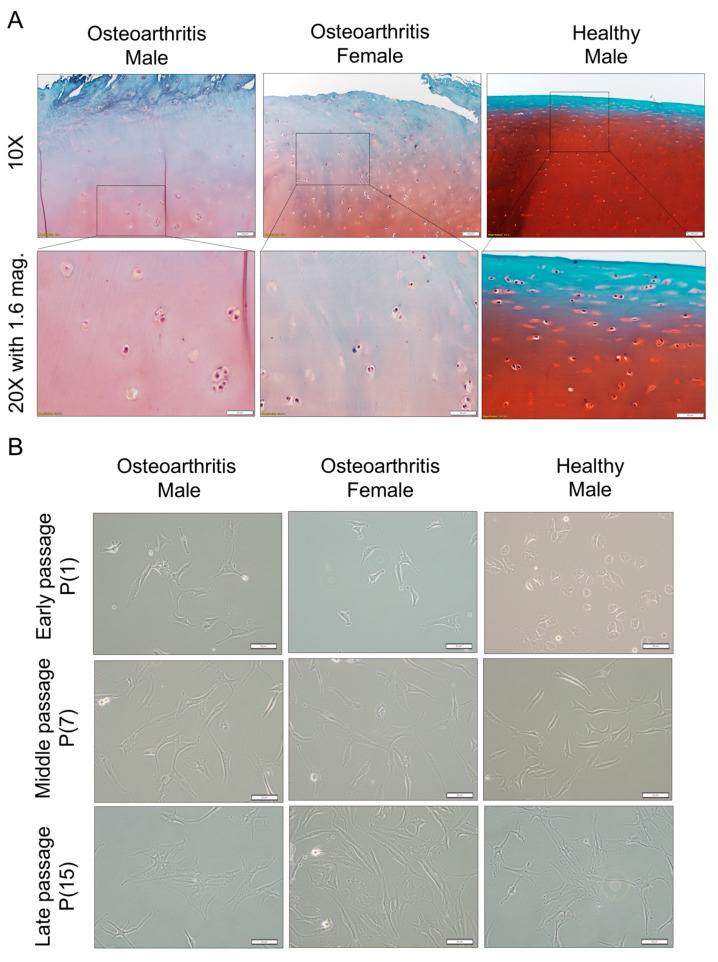
Histological analysis of cartilage samples and effect of serial passaging on cell morphology. (**A**) Safranin O/fast green staining for osteoarthritis, male cartilage; osteoarthritis, female cartilage; healthy male cartilage. Scale bars: 100 µm with 10× objective; 50 µm with 20× objective, 1.6 magnification. (**B**) Cell morphology analysis of early-, middle-, and late-passage chondrocytes from male osteoarthritis, female osteoarthritis, and healthy male. The first horizontal row shows early-passage chondrocytes, the second horizontal row displays middle passage, and the third horizontal row exhibits late-passage chondrocytes. Scale bars: 50 µm with 20× obj.

Safranin O/fast green staining was conducted to assess the extent of ECM degradation in cartilage samples from old and young individuals (Figure 1A). The results revealed greater loss of proteoglycan from osteoarthritic cartilage, with disruption of surface integrity and reduced cellularity. In contrast, the healthy cartilage from a younger individual demonstrated abundant proteoglycan, a smooth surface, and greater cellularity. Chondrocytes from all three subjects reached their Hayflick limit after 30 doublings (15 passages). Early-passage chondrocytes from osteoarthritic donors displayed various cell morphologies, including stellate, elongate, and fibroblastic (Figure 1B).

**Figure 2:**
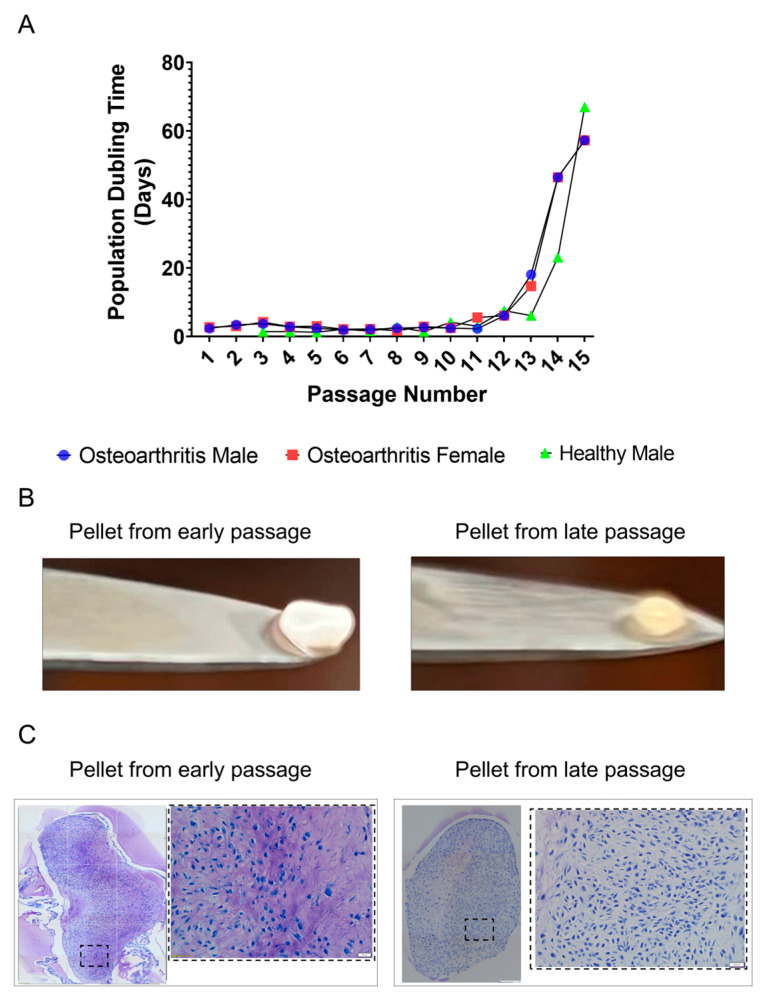
Effect of serial passaging on doubling time and chondrogenesis. (**A**) The population doubling time (days) for cell passages 1 to 15 in osteoarthritis male, female, and healthy male cell populations. (**B**) Effects of replicative senescence on appearance of chondrocyte pellet cultures (**C**) Effects of replicative senescence on toluidine blue staining of chondrocyte pellets. Scale bar: 200 µm with 4× objective and 50 µm with 20× objective.

Early-passage cultures of chondrocytes from the healthy young donor were smaller and comprised a mixture of polygonal and rounded cells. Terminal passage cells were much larger and displayed a mixture of stellate and flattened phenotypes. Passage 7 cells consist of a blend of both early- and late-passage cell types. Although chondrocytes from the healthy donor exhibited morphological differences from those of individuals with OA during early and middle passages, late-passage morphologies were similar in cells from OA and healthy donors. The doubling time of chondrocytes from OA and healthy donors was similar from passage 1 to passage 15 (Figure 2A). Following passage 13, a significant and abrupt increase in doubling time was observed in all cultures regardless of sex or whether the chondrocytes were from normal joints or joints with OA. After reaching passage 15, the cells were left in a T175 flask for a year, during which neither proliferation nor cell death was observed.

Early- and late-passage chondrocytes showed clear differences in their ability to form cartilaginous nodules in pellet culture (Figure 2B). Pellets formed from early-passage chondrocytes were larger and displayed the characteristic white, shiny appearance of cartilage. Those formed from late-passage cells were smaller, with a slight yellow discoloration. The ECM of pellets formed from late-passage chondrocytes lacked the metachromatic staining of pellets formed by early-passage cells (Figure 2C).

### 2.2. DEGs Between OA and Healthy

**Figure 3:**
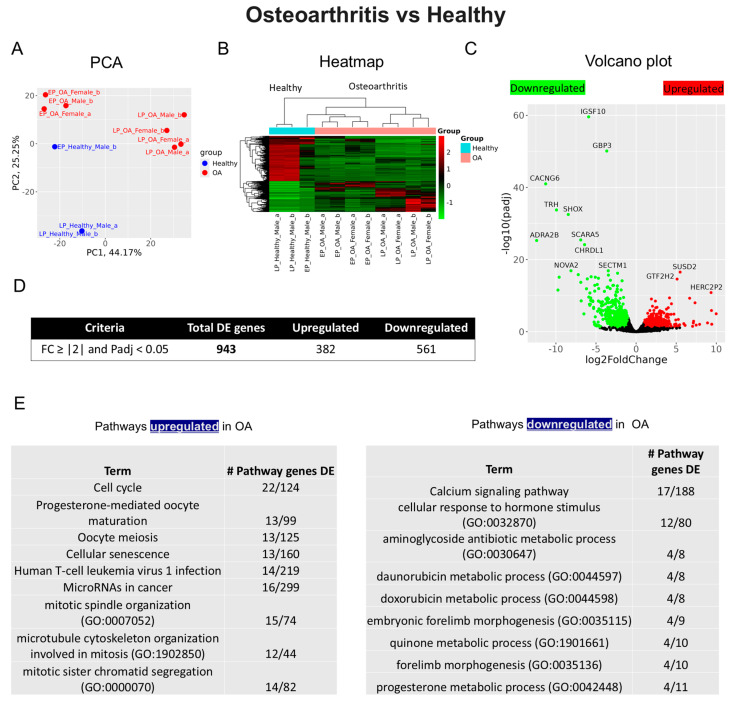
Bulk RNA sequencing analysis of osteoarthritis and healthy group. (**A**) Principal component analysis (PCA) indicating differential expression pattern between osteoarthritic and healthy cells (the early-passage healthy male sample “a” was excluded from the analysis as it was identified as an outlier in comparison to its paired sample “b”). (**B**) Heatmap illustrating upregulated (shown as red) and downregulated (shown as green) genes in the osteoarthritic vs. healthy chondrocytes. (_a, _b) are duplicates from the same sample. (**C**) Volcano plot displaying differences in gene expression between osteoarthritic and healthy chondrocytes. Red dots represent significantly differentially expressed genes (DEGs) that have an absolute fold change (FC) of ≥2, green dots represent genes that have an absolute FC of ≤2, and black dots represent genes not differentially expressed. The FC presented here is the gene expression of osteoarthritis relative to healthy controls. (**D**) Tables showing the most up-/downregulated genes. (**E**) Basic pathway analysis revealing alterations in key pathways linked to aging in osteoarthritis, showing up-/downregulated pathways.

Transcriptomic analysis was conducted to compare gene expression profiles between osteoarthritic and healthy chondrocytes. Principal component analysis (PCA) revealed distinct clustering patterns, demonstrating clear transcriptional separation between OA and healthy groups (Figure 3A). Hierarchical clustering analysis highlighted distinctive gene expression patterns between the two groups (Figure 3B). Differential expression analysis, illustrated by a volcano plot, identified the most significantly up- and downregulated genes (Figure 3C). In total, 943 differentially expressed genes (DEGs) were identified, comprising 382 upregulated and 561 downregulated genes in OA compared to healthy controls (Figure 3D). Subsequent pathway analysis revealed that cellular senescence was among the most significantly upregulated pathways in OA, while the calcium signaling pathway was notably downregulated (Figure 3E).

### 2.3. DEGs Between Early- and Late-Passage Chondrocytes

Transcriptomic profiling was performed on early- and late-passage chondrocyte cultures from all three donors. PCA of all genes showed a clear separation between the late-passage and early-passage chondrocytes, indicating substantial differences in gene expression. DEGs in early- and late-passage cells were then analyzed for each of the three chondrocyte cultures.

**Figure 4:**
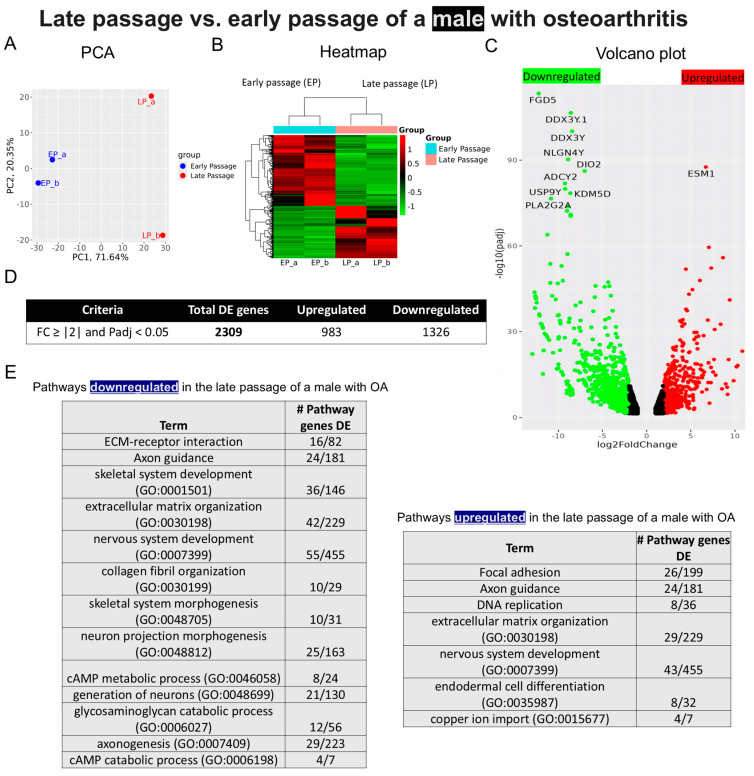
Bulk RNA sequencing analysis of late- and early-passage chondrocytes from a male with osteoarthritis. (**A**) Principal component analysis (PCA) indicating differential expression pattern between late- and early-passage cells. (**B**) Heatmap depicting upregulated (shown as red) and downregulated (shown as green) genes in the late passage vs. early passage of chondrocytes. (_a, _b) are duplicates from the same sample. (**C**) Volcano plot highlighting differences in gene expression between late-passage and early-passage chondrocytes. Red dots represent significantly differentially expressed genes (DEGs) that have an absolute fold change (FC) of ≥2, green dots represent genes that have an absolute FC of ≤2, and black dots represent genes not differentially expressed. The FC presented here is the gene expression of late passages relative to early passages. (**D**) Tables showing up-/downregulated genes. (**E**) Basic pathway analysis revealing alterations in key pathways linked to aging in late passage, showing up-/downregulated pathways.

*Sample 1—Early- and late-passage chondrocytes from a male with OA*: Distinct clusters were observed when comparing the transcriptomes of early- and late-passage chondrocytes obtained from an 80-year-old male with OA (Figure 4A), confirmed by the heat of gene expression patterns (Figure 4B). A volcano plot revealed the most significantly altered genes, with Endothelial Cell-Specific Molecule 1 (*ESM1*) exhibiting the greatest upregulation and *FYVE, RhoGEF*, and PH Domain-Containing Protein 5 (*FGD5*) showing the greatest downregulation (Figure 4C). The analysis identified 2309 DEGs, with 983 genes upregulated and 1326 downregulated in terminal passage chondrocytes (Figure 4D). Pathway analysis revealed downregulation of ECM organization, collagen fibril organization, glycosaminoglycan catabolic process, and skeletal and nervous system development and upregulation of focal adhesion, axon guidance, and, paradoxically, DNA replication genes in late-passage cells (Figure 4E).

**Figure 5:**
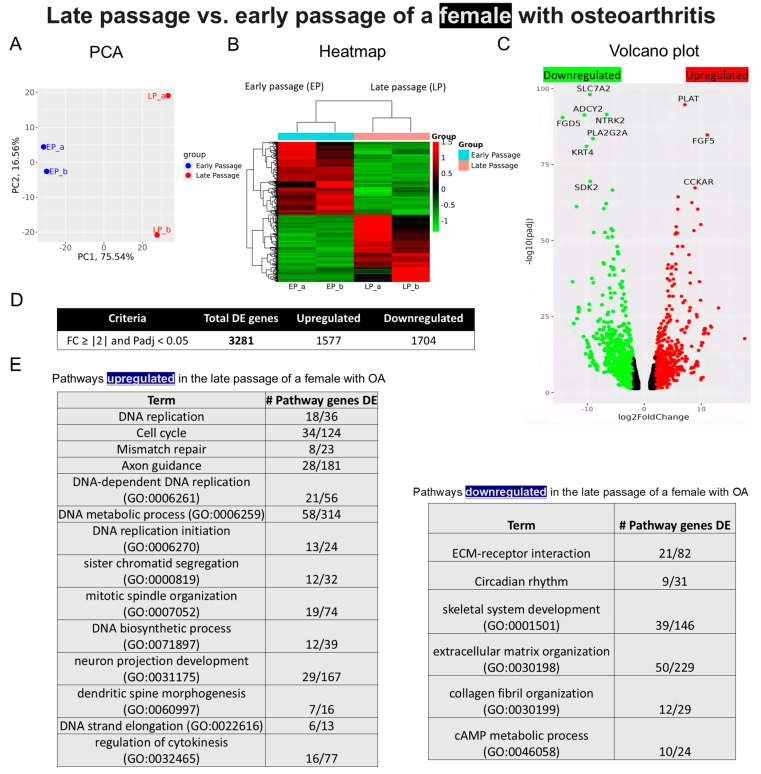
Bulk RNA sequencing analysis of late- and early-passage chondrocytes from a female with osteoarthritis. (**A**) Principal component analysis (PCA) indicating differential expression pattern between late- and early-passage cells. (**B**) Heatmap depicting upregulated (shown as red) and downregulated (shown as green) genes in the late passage vs. early passage of chondrocytes. (_a, _b) are duplicates from the same sample. (**C**) Volcano plot highlighting differences in gene expression between late-passage and early-passage chondrocytes. Red dots represent significantly differentially expressed genes (DEGs) that have an absolute fold change (FC) of ≥2, green dots represent genes that have an absolute FC of ≤2, and black dots represent genes not differentially expressed. The FC presented here is the gene expression of late passages relative to early passages. (**D**) Tables showing up-/downregulated genes. (**E**) Basic pathway analysis revealing alterations in key pathways linked to aging in late passage, showing up-/downregulated pathways.

*Sample 2—Early- and late-passage chondrocytes from a female with OA*: Distinct clusters were also observed when comparing the transcriptomes of early- and late-passage chondrocytes obtained from a 72-year-old female with OA (Figure 5A). This sample displayed a comparable heatmap pattern to that of sample 1 (Figure 5B). The largest gene expression changes were the upregulation of tissue-type plasminogen activator (*PLAT*) and the downregulation of Solute Carrier Family 7 Member 2 (*SLC7A2*) (Figure 5C). A total of 3281 DEGs were identified when comparing early- and late-passage chondrocytes (Figure 5D), with 1577 genes upregulated and 1704 genes downregulated. Similar to sample 1, replicative senescence was associated with the downregulation of genes related to ECM organization and collagen, with upregulation of genes associated with neuron development and, paradoxically, the cell cycle and DNA replication (Figure 5E).

**Figure 6:**
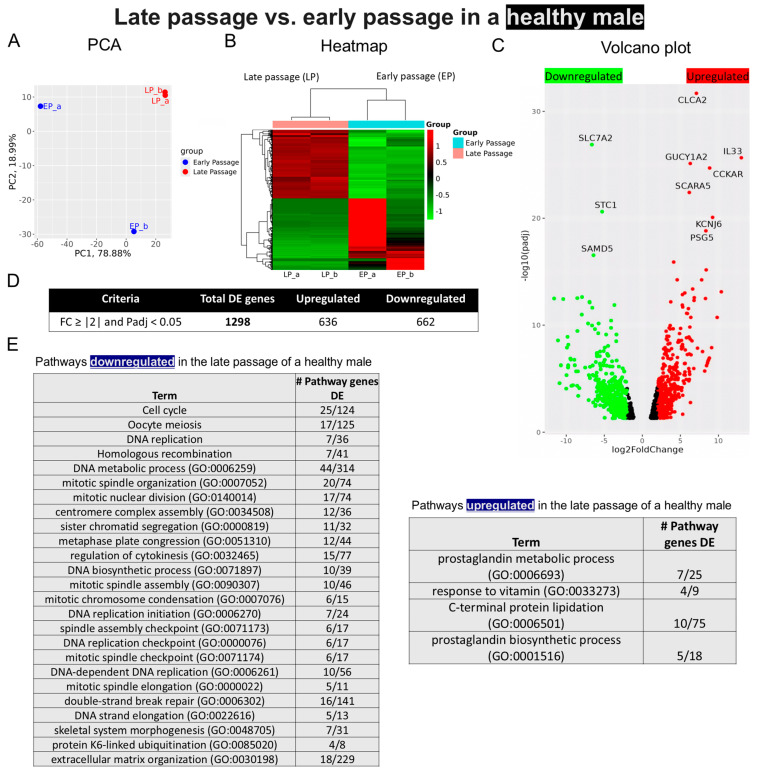
Bulk RNA sequencing analysis of late- and early-passage chondrocytes from a healthy male. (**A**) Principal component analysis (PCA) indicating differential expression pattern between late- and early-passage cells. (**B**) Heatmap depicting upregulated (shown as red) and downregulated (shown as green) genes in the late passage vs. early passage of chondrocytes. (_a, _b) are duplicates from the same sample. (**C**) Volcano plot highlighting differences in gene expression between late-passage and early-passage chondrocytes. Red dots represent significantly differentially expressed genes (DEGs) that have an absolute fold change (FC) of ≥2, green dots represent genes that have an absolute FC of ≤2, and black dots represent genes not differentially expressed. The FC presented here is the gene expression of late passages relative to early passages. (**D**) Tables showing up-/downregulated genes. (**E**) Basic pathway analysis revealing alterations in key pathways linked to aging in late passage, showing up-/downregulated pathways.

*Sample 3—Early- vs. late-passage chondrocytes from a male without OA*: Hierarchical clustering and heatmap patterns differed from those of samples 1 and 2 (Figure 6A,B). The volcano plots highlighted *SLC7A2* as the most downregulated gene, while Chloride Channel Accessory 2 (*CLCA2*) appeared as the most upregulated gene (Figure 6C). Fewer DEGs (1298) were identified in this sample than in samples 1 and 2 (Figure 6D). In contrast to the data from the OA samples, cell cycle and DNA replication genes were downregulated in late-passage cells, while prostaglandin genes were upregulated (Figure 6E).

### 2.4. Analysis of ECM Components, Enzymes That Degrade ECM, and SASP Factors

**Figure 7:**
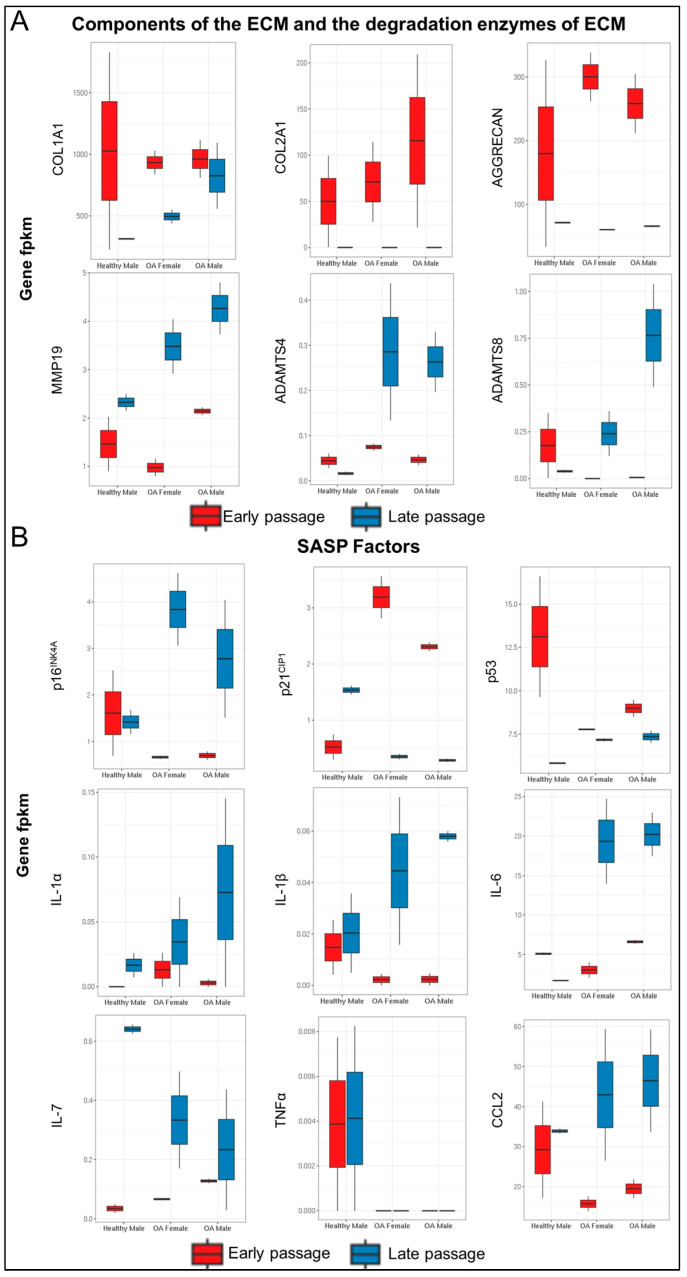
Expression of selected genes related to matrix turnover and senescence during serial passaging. DEGs encompassing (**A**) components of the extracellular matrix (ECM) and enzymes that degrade the ECM and (**B**) genes associated with the senescence-associated secretory phenotype (SASP). Gene fpkm: Fragments Per Kilobase per Million mapped reads.

A focused analysis was undertaken on components of the ECM, the enzymes that degrade them, and SASP factors within each sample (Figure 7). Early-passage cells from all three donors expressed high levels of collagen type I α1 chain (*Col1A1*) as well as cartilage-specific collagen type II α1 chain (*Col2A1*) and *ACAN*. The latter two transcripts fell to extremely low levels in all terminal passage cultures, regardless of donor. The expression of *Col1A1* was more variable, with expression levels dropping to extremely low levels in terminal passage cells from the healthy donor but with expression levels reduced to a lesser degree in cells from osteoarthritic joints (Figure 7A).

Of the enzymes that degrade the ECM of cartilage, expression of *MMP19* was increased in the late-passage chondrocytes of all samples. The expression of *ADAMTS4* and *ADAMTS8* showed significant upregulation in late-passage chondrocytes obtained from OA samples but not in those obtained from healthy cartilage.

The expression of transcripts associated with the SASP (*p16^INK4A^, p21^CIP1^, p53*) was variable. The expression of *p16^INK4A^* did not differ between early- and late-passage cells derived from normal cartilage. The expression in chondrocytes derived from joints with OA was lower than that in chondrocytes from young, healthy cartilage but increased considerably in late-passage cells. The expression of *p21^CIP1^* showed the opposite trend, being higher in early-passage chondrocytes derived from joints with OA than in early-passage cells from young, healthy cartilage but lower in late-passage OA samples than in late-passage samples from young, healthy joints. The expression of *p53* decreased with passage to varying degrees in all cultures (Figure 7B).

Among the cytokine components of SASP, *IL-1α* expression increased in all cultures with passage. The expression of *IL-1β* did not increase with the passage of chondrocytes derived from young, healthy joints but increased in chondrocytes derived from OA cartilage. While *IL-6* expression was roughly similar in early-passage cells of all sources, it increased considerably with the passage of chondrocytes derived from joints with OA but fell in chondrocytes derived from healthy joints. *IL-7* expression increased in all late-passage samples. *TNFα* expression was barely detectable in any culture. C-C motif chemokine ligand 2 (*CCL2*) was upregulated in the late passage of chondrocytes derived from patients with OA but not those derived from healthy cartilage.

### 2.5. Pathway Analysis Comparing Chondrocytes from a Male and Female with OA

**Figure 8:**
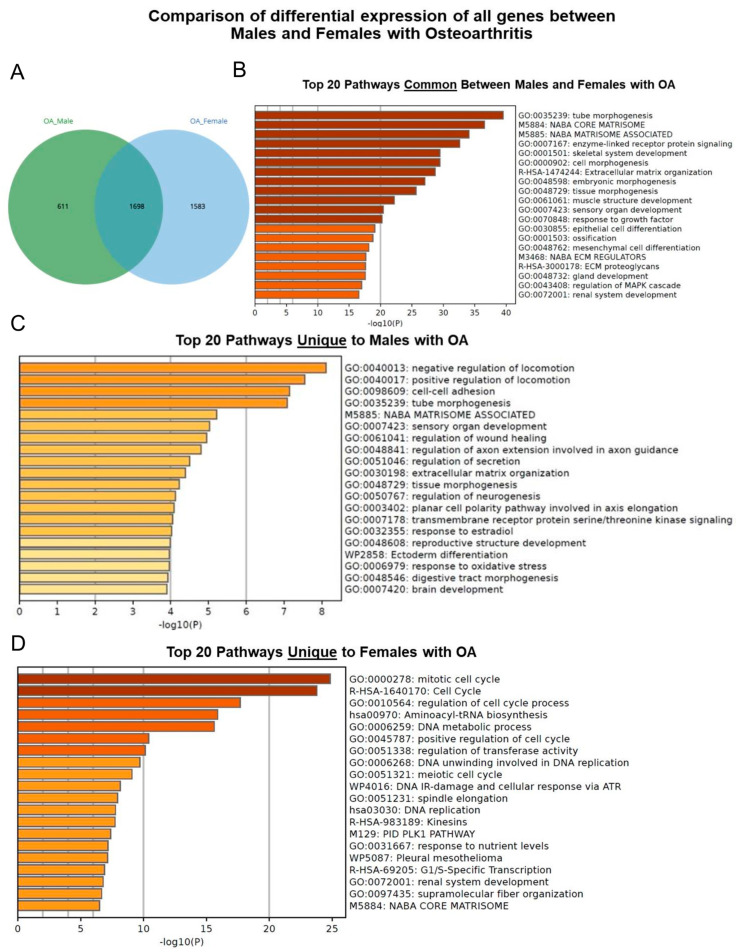
Basic pathway analysis of differentially expressed genes in chondrocytes from males and females with osteoarthritis. (**A**) Venn diagram illustrating shared and distinct genes in males and females. (**B**) Top 20 pathways common between males and females with OA. (**C**) Top 20 pathways unique to males with OA. (**D**) Top 20 pathways unique to females with OA. Shades of orange indicate the most significantly regulated pathways, with darker shades representing higher levels of regulation and lighter shades indicating lower levels.

Pathway analysis was conducted to compare the DEG profiles between chondrocytes derived from the male and female samples with OA, utilizing data from samples 1 and 2. This analysis revealed 611 genes unique to males and 1583 genes unique to females, with 1698 genes common between the sexes (Figure 8A). The top 20 pathways common between males and females with OA included ECM organization, collagen formation, musculoskeletal development, and nervous system development (Figure 8B). The top 20 pathways unique to males with OA encompassed ECM organization, mitotic cell cycle, and regulation of neuron differentiation (Figure 8C). The top 20 pathways unique to females with OA were featured in the regulation of the cell cycle process, DNA metabolic process, and Polo-Like Kinase 1 (PID-PLK1) pathway (Figure 8D).

### 2.6. Quantitative RT-PCR

**Figure 9:**
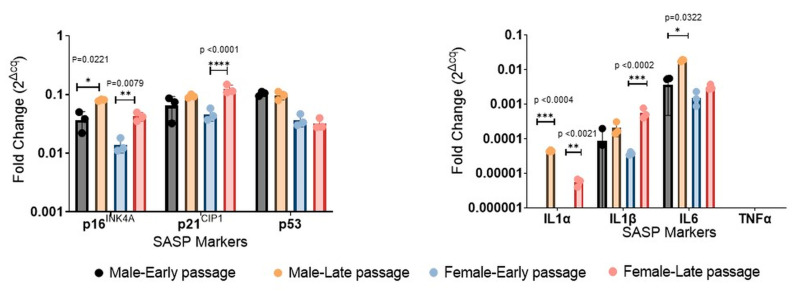
Measurement of key transcripts by qRT-PCR. Quantification *of IL-1α*, *IL-1β*, *IL-6*, *p16^INK4A^, p21^CIP1^*, and *p53* using qRT-PCR (* *p* < 0.05; ** *p* < 0.01; *** *p* < 0.001; **** *p* < 0.0001).

Certain findings from bulk RNA sequencing were validated by qRT-PCR (Figure 7). Consistent with the RNA sequencing data, significant upregulation of *p16^INK4A^*, *IL-1α*, *IL-1β*, and *IL-6* was observed in late-passage chondrocytes (Figure 9).

## 3. Discussion

In this study, we demonstrated that serial passaging of chondrocytes to replicative senescence induced characteristic morphological changes and reduced their chondrogenic potential. Chondrocytes reached replicative senescence at passage 15 after 30 population doublings, which is consistent with earlier data [32]. It is remarkable that chondrocytes derived from old (72 years, 80 years) and young (26 years) donors had the same Hayflick limit, as diploid cells from young individuals typically have a greater mitotic potential than those from old individuals. Indeed, the pattern of replicative senescence was remarkably similar between the three cultures. It is possible that the quiescent state of articular chondrocytes in vivo preserves their mitotic potential as the person ages chronologically. Consistent with this, Rose et al. [40] demonstrated that although DNA damage occurs in osteoarthritic chondrocytes, there is no evidence of telomere shortening. Nevertheless, when plated into monolayer culture, chondrocytes from the two old donors already had the morphology of late-passage cells. Because this is a small pilot study involving only three donors, it is not possible to draw strong, general conclusions, but these observations are nevertheless intriguing. We also showed that late-passage chondrocytes have a reduced capacity to form cartilaginous tissues, as evidenced by smaller pellet sizes and decreased proteoglycan content. This confirms that replicative senescence impairs the chondrogenic potential of cultured chondrocytes.

Bulk RNA sequencing analysis revealed significant differences between OA and healthy control groups, identifying 382 upregulated and 561 downregulated genes. Volcano plot analysis demonstrated the most significantly altered genes in OA samples. For instance, immunoglobulin superfamily member 10 (*IGSF10*), which is thought to play a role in bone remodeling, was significantly altered in early OA development [41]. Similarly, guanylate-binding protein 3 (*GBP3*) showed marked downregulation in facet joint OA [42]. The calcium signaling pathway emerged as one of the most downregulated pathways in our study, supported by the decreased expression of calcium voltage-gated channel auxiliary subunit gamma 6 (*CACNG6*). This downregulation may impact calcium channel activity, potentially affecting chondrocyte survival, matrix production, and mechanotransduction responses [38]. These findings align with previous research comparing gene expression profiles in knee joint tissues from young and aged mice with experimentally induced OA [43]. This earlier study, using microarray analysis, revealed significant age-related differences in calcium signaling genes, including *CACNG6*. Furthermore, the upregulation of the cellular senescence pathway in OA samples was an expected finding, consistent with the known role of cellular aging in OA pathogenesis [43].

Notably, our analysis also revealed the downregulation of thyrotropin-releasing hormone (*TRH*) in OA samples. This finding has important implications, as thyroid hormone plays a crucial role in endochondral ossification and regulates the expression of genes controlling chondrocyte maturation and matrix synthesis [44,45]. Thyroid hormone deficiency leads to decreased calcitonin secretion, disrupting calcium homeostasis and bone metabolism, which ultimately contributes to OA development [46]. This relationship is further supported by clinical evidence demonstrating that thyroid-stimulating hormone (*TSH*) levels show a significant positive correlation with both the prevalence and progression of OA in patients with autoimmune thyroid disease [47].

Additionally, bulk RNA sequencing demonstrated clear separation between early- and late-passage cells, highlighting substantial differences in gene expression. In several cases, changes in gene expression were common in all cultures. The uniform decline in expression of *Col2A1* and *ACAN* core protein with passage, for instance, confirms loss of the chondrocyte phenotype as the cells undergo replicative senescence. Other changes common to all cultures included the upregulation of *IL-1α*, *IL-7*, and *MMP19* with passage, as well as the near absence of *TNF-α* in any culture. It is intriguing, however, that the changes in gene expression as a result of replicative senescence were not identical between chondrocytes recovered from normal joints and those recovered from joints with OA. Indeed, changes with passage in the expression of several genes, including *p16^INK4A^*, *p21^CIP1^*, *IL-1β*, *IL-6*, *ADAMTS4*, and *ADAMTS8*, while qualitatively similar between the two samples from OA cartilage, differed from changes with passage in cells derived from healthy cartilage. Moreover, pathway analysis revealed that the expression of genes associated with DNA replication was paradoxically upregulated in late-passage chondrocytes derived from OA cartilage but as might be expected, downregulated in late-passage cells derived from healthy cartilage. Collectively, these data are consistent with the induction of epigenetic changes occurring in situ as a result of aging and OA that survive passaging.

The preliminary nature of this study does not justify a deep analysis of the totality of the bulk RNA sequencing data, but several findings are worth mentioning. For instance, volcano plots revealed that *FGD5* was the most downregulated gene in late-passage OA chondrocytes. While the precise involvement of *FGD5* in OA development is not known, Yang et al. [48] demonstrated that *FGD5-AS1* regulates OA progression via the *miR-302d-3p/TGFBR2* axis, protecting chondrocytes from inflammation-induced injury and reducing the ECM degradation. Phospholipase A2 Group IIA (*PLA2G2A*) expression was also downregulated in late passages of both male and female OA chondrocytes. In agreement with this finding, Tsolis et al. [49] demonstrated that *PLA2G2A* exhibits higher expression in healthy chondrocytes compared to chondrocytes from OA cartilage. Likewise, adenylyl cyclase type 2 (*ADCY2*) was also downregulated in late-passage cultures derived from both male and female subjects with OA. Notably, *ADCY2* was undetectable in healthy chondrocytes [40].

Concerning possible sex differences in gene expression with replicative senescence, Deiodinase 2 (*DIO2*) was downregulated in late-passage male OA chondrocytes. This gene is recognized as one of several OA susceptibility genes that play a crucial role in pathways involved in both pre- and post-natal joint development, ultimately contributing to endochondral ossification [50]. Similarly, genes located in the male-specific region of the Y chromosome, namely DEAD-Box Helicase 3, Y-Linked (*DDX3Y*), Ubiquitin-Specific Peptidase 9, Y-Linked (*USP9Y*) and Neuroligin 4, and Y-Linked (*NLGN4Y*), were found to be significantly downregulated in a late-passage male OA sample, an observation consistent with the data of Liu et al. [51]. Also of possible relevance to sex-related differences in the incidence of OA, *PLAT*, an estrogen-regulated gene [52], was upregulated in the late-passage female OA sample.

Basic pathway analyses of late passages were performed to identify sex-specific DEGs in OA. The top 20 pathways common to both males and females with OA were generally related to ECM organization, collagen formation, musculoskeletal development, and nervous system development. However, females (1583 genes) showed distinct pathways compared to males (611 genes), including regulation of cell cycle processes, DNA metabolism, and the PLK1 signaling pathway. PLK1 is a key regulator of mitotic cell division and has been shown to be overexpressed in several human cancers, including breast cancer [53]. Driscoll et al. [54] showed that inhibiting PLK1 causes post-mitotic DNA damage and senescence in tumor cell lines. Kim et al. [55] found that decreasing PLK1 levels stimulates senescence through a *p53*-dependent pathway. Additionally, Wierer et al. [56] showed that PLK1 mediates estrogen receptor-regulated genes in human breast cancer cells, suggesting PLK1 could play a role in the sex differences seen in the incidence of OA.

The small sample size is the main limitation of this pilot study. Additional studies with more subjects from both sexes are important to fully characterize chondrocyte senescence and elucidate sex differences in relation to its pathophysiologic role in OA. The limited availability of normal cartilage specimens is a major obstacle to achieving this expeditiously. Another consideration is collecting samples from individuals with idiopathic, rather than post-traumatic, OA, where chondrocyte senescence may be the key underlying pathophysiologic process. Overall, while preliminary, these data provide insights into transcriptomic changes underlying chondrocyte aging and OA development. They also question whether replicative senescence of chondrocytes in vitro is an appropriate surrogate for in situ chondrocyte senescence when studying its relationship to the etiopathophysiology of OA.

## 4. Materials and Methods

### 4.1. Study Design

Osteoarthritic cartilage was obtained from patients diagnosed with moderate to severe OA, confirmed as Kellgren–Lawrence grade 3 or 4, during total knee arthroplasty in the Department of Orthopedic Surgery at Mayo Clinic. Eligible donors included 80-year-old male and 72-year-old female. Healthy cartilage sample was obtained from a 26-year-old male with no history of joint disease or injury. The cartilage showed no signs of degeneration, confirmed through histological analysis.

Chondrocytes recovered from knee articular cartilage were serially passaged until they reached their Hayflick limit. RNA was extracted from first passage and terminal passage cultures and subjected to bulk sequencing and bioinformatic analysis. The abundance of certain transcripts was confirmed by quantitative RT-PCR. The quality of cartilage formed in pellet culture was assessed at passage 1 and passage 15 using chondrocytes from the female donor.

### 4.2. Biospecimen Processing

#### 4.2.1. Cartilage Collection

Human osteoarthritic cartilage biopsies (*n* = 2, patient 1: male aged 80; patient 2: female aged 72) were collected from individuals undergoing total knee arthroplasty at Mayo Clinic, Department of Orthopedic Surgery with approval by the Institutional Review Board.

#### 4.2.2. Chondrocyte Isolation

Full-thickness pieces of articular cartilage were removed aseptically from the femoral condyles and tibial plateaux using a scalpel. The cartilage was cut into 1–2 mm^3^ pieces and washed with phosphate-buffered saline (Gibco, Gaithersburg, MD, USA). The cartilage fragments were digested for 2 h at 37 °C in 0.2% pronase (Millipore Sigma, St. Louis, MO, USA) in Dulbecco’s Modified Eagle Medium/Nutrient Mixture F-12 (DMEM/F-12; Sigma-Aldrich, St. Louis, MO, USA) with a mixture of penicillin (100 U/mL) and streptomycin (100 µg/mL) (1% P/S, Gibco, Gaithersburg, MD, USA). After pronase digestion, the cartilage fragments were further digested for 16 h at 37 °C in 0.05% type II collagenase (Gibco, Gaithersburg, MD, USA) in DMEM/F-12 media supplemented with 5% fetal bovine serum (FBS; Gibco, Gaithersburg, MD, USA) and 1% P/S. The digest was filtered through a 70 µm cell strainer, and cells were recovered and washed. Cells were counted, seeded into 75 cm^2^ culture flasks at a density of 1.0 × 10^4^ cells/cm^2^ in 10 mL of DMEM/F12 supplemented with 1% P/S and 10% FBS, and incubated at 37 °C in 5% CO_2_. Human articular chondrocytes from a normal knee joint were purchased from Lonza (Basel, Switzerland) (*n* = 1, male aged 26).

### 4.3. Serial Passage of Chondrocytes

Cells were recovered from confluent cultures by trypsinization and re-seeded into 75 cm^2^ flasks at a 1:4 split ratio such that each passage produced 2 population doublings. Briefly, cells were treated with 4 mL of TrypLE™ (Thermo Fisher Scientific, Waltham, MA, USA) and incubated at 37 °C in 5% CO_2_ for 5 min to detach the cells. Subsequently, 8 mL of medium containing 10% FBS was added, followed by centrifugation at 400× *g* for 5 min. The supernatant was discarded, and the pellet was reconstituted in 1 mL of medium. Cells were counted, and 250 µL of the cell suspension was seeded into a 75 cm^2^ flask with 10 mL medium. This process was repeated until cells could no longer achieve confluence, at which point they were considered to have reached their Hayflick limit. Doubling time was calculated from the following formula: Doubling time = [Duration⋅*ln*(2)]/[*ln*(Final concentration/Initial concentration)] [57].

### 4.4. Three-Dimensional Pellet Culture

First passage or terminal passage chondrocytes from the female donor were recovered and re-cultured as pellets as previously described [58]. Briefly, cells were plated at a density of 2 × 10^5^ cells per well in polypropylene, v-bottom, 96-well plates (Corning, Corning, NY, USA). Plates were centrifuged at 400× *g* for 5 minutes; supernatants were removed and replaced with chondrogenic medium (high glucose DMEM (Sigma-Aldrich, St. Louis, MO, USA), 1% insulin–transferrin–selenium premix (BD Biosciences, Franklin Lakes, NJ, USA), 100 nM dexamethasone (Sigma-Aldrich, St. Louis, MO, USA), 50 µg/mL ascorbate-2-phosphate (Sigma-Aldrich, St. Louis, MO, USA), 1 mM sodium pyruvate (Sigma-Aldrich, St. Louis, MO, USA), 40 µg/mL L-proline (Sigma-Aldrich, St. Louis, MO, USA), and 10 ng/mL *TGFβ1* (PeproTech, Cranbury, NJ, USA). During the initial 24 hours of incubation, cells formed free-floating aggregates. Media were changed the following morning and every other day thereafter.

### 4.5. Histology

#### 4.5.1. Histological Preparation of Human Knee Femoral Condyle and Sectioning

Cartilage specimens from OA donors were fixed in 10% neutral-buffered formalin for 72 h. For comparison, healthy cartilage from a different donor was included, as these healthy chondrocytes were purchased from a commercial source. The fixed biospecimens were then dehydrated through a graded ethanol series and embedded in paraffin. Using an automated microtome (HM 355S, Thermo Fisher Scientific, Kalamazoo, MI, USA), 5-μm sections were cut and mounted onto positively charged microscope slides (Superfrost™ Plus Microscope Slides, Fisher Fischer Scientific; Pittsburgh, PA, USA).

#### 4.5.2. Hematoxylin/Eosin and Safranin-O/Fast Green Staining

Standard safranin O/fast green staining protocol was followed for paraffin-embedded tissue sections. Briefly, 5 μm sections were deparaffinized and rehydrated through xylenes and graded alcohols. Weigert’s iron hematoxylin (Electron Microscopy Sciences, Hatfield, PA, USA) was applied for 10 min, followed by 0.4% aqueous fast green (Sigma-Aldrich, St. Louis, MO, USA) for 4 min and 0.125% safranin O (Electron Microscopy Sciences, Hatfield, PA, USA) for 5 min. Sections were quickly dehydrated through ethyl alcohols, cleared in xylene, and mounted in xylene-based medium. Bright-field images were obtained using an automated inverted microscope (Olympus IX83 microscope, Waltham, MA, USA).

#### 4.5.3. Pellet Cultures

Pellets were fixed for 1 h in 4% paraformaldehyde, centrifuged at 400 rpm for 5 min, washed with 95% alcohol, and resuspended in 100 µL of 0.8% low-melting-point agarose solution (Invitrogen, Carlsbad, CA, USA), followed by re-centrifugation at 1500 rpm for 5 min. The resulting agarose-embedded cell pellet was allowed to solidify for 30 min at 4 °C, then processed as per routine histology; 5 µm thick sections were cut using an automatic microtome (HM 355S, Thermo Fischer Scientific, Kalamazoo, MI, USA) and mounted onto positively charged slides (Superfrost™ Plus Microscope Slides, Thermo Fisher Scientific, Kalamazoo, MI, USA).

#### 4.5.4. Toluidine Blue Staining

Sections were deparaffinized and rehydrated through a series of xylenes and graded alcohols to distilled water, followed by staining with 0.1% toluidine blue pH 2.3 for 1.5 min, rinsing in distilled water for 1 min, then quickly dehydrating in a graded ethyl alcohol series, cleared with xylene, and mounted with xylene-based mounting medium (Richard-Allan Scientific™, Kalamazoo, MI, USA). Bright-field images were acquired using an automated inverted microscope (Olympus IX83, Center Valley, PA, USA) at 4× and 20× magnifications using the cellSens Olympus imaging software, version V4.1.1 (available at: https://www.olympus-lifescience.com/en/downloads/detail-iframe/?0[downloads][id]=847253977, accessed on 18 May 2015).

### 4.6. Bulk RNA Sequencing and Data Analysis

#### 4.6.1. RNA Isolation

A two-step RNA isolation protocol was followed. Organic phase isolation was performed with TriReagent^®^/bromo–3–chloropropane (BCP) (Thermo Fisher Scientific/Molecular Research Center, Inc., Cincinnati, OH, USA) and then used for total RNA isolation using an RNeasy Plus Mini Kit (Qiagen, Germantown, MD, USA) according to the manufacturer’s instructions. RNA concentration was measured using a nanophotometer^®^ (Implen, Inc., Westlake Village, CA, USA). RNA purity was confirmed by spectrophotometry (A_260/280_ and A_260/230_ > 2.0). RNA integrity was assessed using an Agilent 2100 bioanalyzer (Agilent, Santa Clara, CA, USA) to confirm that samples had an RNA integrity number (RIN) > 8.

#### 4.6.2. Illumina Stranded mRNA Library Preparation and Illumina NextSeq2000 Sequencing

RNA libraries were prepared using 200 ng of total RNA in a volume of 20 µL according to the manufacturer’s instructions for the Illumina Stranded mRNA Ligation Sample Prep Kit (Illumina, San Diego, CA, USA). The concentration and size distribution of the completed libraries were determined using an Agilent Bioanalyzer DNA 1000 chip and Qubit fluorometry (Invitrogen, Carlsbad, CA, USA). Libraries were sequenced using Illumina’s standard protocol for the NovaSeq 6000 (Illumina, Inc., San Diego, CA, USA). The NovaSeq SP PE100 flow cell was sequenced as 100 × 2 paired-end reads using NovaSeq Control Software version v1.8.0 (available at: https://support.illumina.com/downloads/novaseq-control-software-v1-8.html, accessed on 5 April 2023). Base-calling was performed using Illumina’s RTA version 3.4.4.

#### 4.6.3. Bioinformatics and Data Analysis

Fastq files for all samples were processed using the Mayo Clinic RNA-Seq bioinformatics pipeline, MAP-RSeq version 3.1.4 (available at: https://bioinformaticstools.mayo.edu/research/maprseq/, accessed on 27 June 2014) [59]. Gene and exon expression quantification was performed using the Subread [60] package to obtain both raw gene counts and normalized Fragments Per Kilobase per Million mapped reads (FPKM) values. Finally, FASTQC, version 0.12.0 (available at http://www.bioinformatics.babraham.ac.uk/projects/fastqc, accessed on 29 June 2007) [61] and MultiQC, version v1.16 (available at: http://multiqc.info, accessed on 13 September 2023) [62] tools were used for comprehensive quality control assessment of the aligned reads. PCA and hierarchical clustering were performed for quality control, exploratory data analysis, and to identify sample outliers. Using the raw gene counts generated from the MAP-RSeq pipeline, genes with significant differential expression between different samples were assessed using edgeR, version 4.3.1 (available at: https://bioconductor.org/packages/release/bioc/html/edgeR.html, accessed on 14 October 2020) [63]. Genes with counts per million ˂ 0.5 were filtered out prior to DEG analysis. Initial transcriptomic analysis was performed to identify DEGs between osteoarthritic and healthy chondrocytes, independent of passage number. Subsequently, three comparisons were performed, including (i) late-passage vs. early-passage chondrocytes from males with OA, (ii) late-passage vs. early-passage chondrocytes from females with OA, and (iii) late-passage vs. early-passage chondrocytes from the healthy male. DEGs were visually represented using volcano plots and heatmaps generated with the R package, version 4.3.1 (available at: https://cran.r-project.org/bin/windows/base/old/4.3.1/, accessed on 16 June 2023) and ggplot2, version 3.4.3 (available at: https://ggplot2.tidyverse.org/, accessed on 14 August 2023). DEGs were reported along with their magnitude of change (log2 fold change ≥ 1 or ≤−1) and level of significance (False Discovery Rate (FDR) < 0.05). Pathway analysis was conducted with R package enrichR, version 3.2 (available at: https://maayanlab.cloud/Enrichr/, accessed on 13 April 2023) [64] or Metascape, version 3.5 [65] (available at https://metascape.org, accessed on 1 January 2020) using human pathway settings.

### 4.7. Quantitative Real-Time Polymerase Chain Reaction (qRT-PCR)

Total RNA was isolated from samples snap-frozen in liquid nitrogen and homogenized in an Eppendorf tube using 3 mm Tungsten carbide beads and a TissueLyser II system (Qiagen, Germantown, MD, USA) set to 4 min at 30 Hz; 1 µg of cDNA was synthesized using a high-capacity cDNA reverse transcription kit (Applied Biosystems, Waltham, MA, USA). PCR experiments were performed using 20 ng of cDNA per reaction, qRT-PCR Brilliant III ultra-fast master mix kit (Agilent, Santa Clara, CA, USA), and TaqMan gene expression probes (Thermo Fisher Scientific, Kalamazoo, MI, USA) as follows: *IL-1α* (Hs00174092_m1), *IL-1β* (Hs01555410_m1), *IL-6* (Hs00174131_m1), *p16^INK4A^* (Hs00923894_m1), *p21^CIP1^* (Hs00355782_m1), and *p53* (Hs01034249_m1). The PCR assay was conducted using the AriaMx qRT-PCR detection system (Agilent, Santa Clara, CA, USA). Gene expression values were normalized to the *GAPDH* housekeeping gene to calculate the Δ^Ct^ value.

### 4.8. Statistical Analysis

Statistical analyses for qRT-PCR used GraphPad Prism 9.2.0 (GraphPad, Boston, MA, USA). The experimental design involved 3 biological replicates (*n* = 3). Data distribution was assessed using either the Kolmogorov–Smirnov test or Shapiro–Wilk test. A two-tailed unpaired *t*-test or two-way ANOVA was employed for normally distributed data, while the Mann–Whitney U-test was utilized for non-normally distributed data. The results were presented as mean ± standard deviation (SD). Statistical significance was set at a *p*-value < 0.05.

## 5. Conclusions

In this small (*n* = 3) pilot study, distinct DEG differences were observed between early- and late-passage chondrocytes, as well as between males and females and those with or without OA. These findings indicate possible important age- and sex-related differences in gene expression associated with OA. The upregulation of *IL-1α*, *IL-1β*, *IL-6*, *IL-7*, and *p16^INK4a^* in late-passage chondrocytes from OA samples highlights the link between cellular senescence and inflammation. This was associated with significant downregulation of key ECM genes encoding *Col2A1* and *ACAN* core protein, along with upregulation of matrix-degrading enzymes in late-passage chondrocytes derived from joints with OA, consistent with a pivotal role of chondrocyte senescence in OA pathogenesis. Additionally, the sample derived from a female patient exhibited DEG patterns that differed from those of samples derived from males, although more samples are needed to confirm a sex-related difference in DEG. In several instances, transcriptomic changes with the passage of chondrocytes from joints with OA were different from those occurring with the passage of normal chondrocytes.

## Data Availability

All computational analysis pipelines can be found on NCBI Gene Expression Omnibus (GEO) (https://www.ncbi.nlm.nih.gov/geo/, accessed on 6 November 2023) under accession number GSE246425.

## Abbreviations

*ACAN*	Aggrecan
*ADAMTS*	A Disintegrin and Metalloproteinase with Thrombospondin Motifs
*ADCY2*	Adenylyl Cyclase Type 2
*CACNG6*	Calcium Voltage-Gated Channel Auxiliary Subunit Gamma 6
*CCL2*	C-C Motif Chemokine Ligand 2
*CLCA2*	Chloride Channel Accessory 2
*Col1A1*	Collagen Type I Alpha 1 Chain
*Col2A1*	Collagen Type II Alpha 1 Chain
*DDX3Y*	DEAD-Box Helicase 3, Y-Linked
DEG	Differentially Expressed Gene
*DIO2*	Deiodinase 2
DMEM/F-12	Dulbecco’s Modified Eagle Medium/Nutrient Mixture F-12
ECM	Extracellular Matrix
*ESM1*	Endothelial Cell Specific Molecule 1
FBS	Fetal Bovine Serum
FC	Fold Change
*FGD5*	FYVE, RhoGEF and PH Domain-Containing 5
*GBP3*	Guanylate-Binding Protein 3
GM-CSF	Granulocyte Macrophage-Colony Stimulating Factor
*IGF-1*	Insulin-Like Growth Factor-1
*IGSF10*	Immunoglobulin Superfamily Member 10
*IL-1α*	Interleukin-1 Alpha
*IL-1β*	Interleukin-1 beta
*IL-6*	Interleukin-6
*IL-7*	Interleukin-7
*MMP*	Matrix Metalloproteinase
*NLGN4Y*	Neuroligin 4, Y-Linked
OA	Osteoarthritis
*OSM*	Oncostatin M
P/S	Penicillin-Streptomycin
*p16^INK4A^*	Cyclin-Dependent Kinase Inhibitor 2A
*p21^CIP1^*	Cyclin-Dependent Kinase Inhibitor 1A
*P53*	Tumor protein p53
PCA	Principal Component Analysis
PID-PLK1	Polo-Like Kinase 1
*PLA2G2A*	Phospholipase A2 Group IIA
*PLAT*	Tissue-Type Plasminogen Activator
ROS	Reactive Oxygen Species
RT-PCR	Reverse Transcription Polymerase Chain Reaction
SASP	Senescence-Associated Secretory Phenotype
SA-β-Gal	Senescence-Associated β-Galactosidase
*SLC7A2*	Solute Carrier Family 7 Member 2
*TGF-β*	Transforming Growth Factor-Beta
*TNF-α*	Tumor Necrosis Factor-alpha
*TRH*	Thyrotropin-Releasing Hormone
*TSH*	Thyroid-Stimulating Hormone
*USP9Y*	Ubiquitin Specific Peptidase 9, Y-Linked
